# Tribulus terrestris Ameliorates Oxidative Stress-Induced ARPE-19 Cell Injury through the PI3K/Akt-Nrf2 Signaling Pathway

**DOI:** 10.1155/2020/7962393

**Published:** 2020-07-28

**Authors:** Zhenli Yuan, Weiwei Du, Xiangdong He, Donglei Zhang, Wei He

**Affiliations:** ^1^The School of Pharmacy, He University, Shenyang 110163, China; ^2^Shenyang Industrial Technology Institute of Ophthalmology, Shenyang 110163, China

## Abstract

Oxidative stress on retinal pigment epithelial (RPE) cells has been confirmed to play a crucial role in the development and progression of age-related macular degeneration (AMD) or other retinal degenerative diseases. Tribulus terrestris (TT) is a Chinese traditional herb medicine, which has been used for the treatment of ocular diseases for many centuries. In this study, we investigated the underlying mechanisms of TT and examined its ability to protect and restore the human retinal pigment epithelial cells (ARPE-19) against H_2_O_2_-induced oxidative stress. Our data show that 200 *μ*g/mL of ethanol extract of Tribulus terrestris (EE-TT) significantly increased the cell viability and prevented the apoptosis of H_2_O_2_-treated ARPE-19 cells through the regulation of Bcl2, Bax, cleaved caspase-3, and caspase-9. Treatment with EE-TT also significantly decreased the upregulated reactive oxygen species (ROS) activities and increased the downregulated superoxide dismutase (SOD) activities induced by H_2_O_2_ in ARPE-19 cells. Additionally, H_2_O_2_ at 1 mM significantly decreased the mRNA expression levels of Nrf2, CAT, SOD1, SOD2, HO-1, GST-pi, NQO1, and GLCM in ARPE-19 cells; however, treatment with EE-TT reversed the downregulated mRNA expression levels of all these genes induced by H_2_O_2_. Furthermore, treatment with 200 *μ*g/mL EE-TT alone for 24 h significantly increased Nrf2, HO-1, NQO1, and GCLM mRNA expressions in ARPE-19 cells when compared with untreated control cells. Pretreatment with the inhibitor of PI3K/Akt signaling (LY294002) completely blocked these EE-TT-upregulated mRNA expressions and abolished the improvement of cell viability in H_2_O_2_-treated ARPE-19 cells. These findings all suggest that Tribulus terrestris has significant antioxidant effects on oxidative stressed ARPE-19 cells through regulating PI3K/Akt-Nrf2 signaling pathway.

## 1. Introduction

Numerous studies have shown that visual impairment caused by retinal damage is among one of the most important causes of blindness, in particular, pathological retinal pigment epithelial cell layer damage in age-related macular degeneration (AMD) and other retinal diseases (such as diabetes and retinitis pigmentosa) [1, 2]. Therefore, protecting RPE cells from injury or delaying RPE cell damage is important in AMD treatment.

The human retinal pigment epithelial (RPE) is a monolayer of pigmented cells lying in the interface between the photoreceptors of the neurosensory retina and the choroidal capillary bed; it constitutes the outer blood-retinal barrier preventing the entrance of toxic molecules and plasma components into the retina [3]. The RPE cell layer plays an important role due to its unique location, which enables it to protect fundus tissue against photooxidation, as well as its function that allows it to process visual cycle [4]. Under normal physiological conditions, the retina demands higher oxygen supply against the high levels of cumulative irradiation surrounding the retinal, which render RPE cells vulnerable to oxidative damage. Reactive oxygen species (ROS) induced by oxidative stress is the main cellular reactive oxygen intermediates, which include free radicals, hydrogen peroxide, and oxygen ion from the byproducts of oxygen metabolism, inducing cell damage and apoptosis in the different cell types [5, 6]. In normal cells, an accurate balance between ROS generation and antioxidant systems maintains cellular redox homeostasis. The cellular antioxidant systems include the enzymatic antioxidants and the nonenzymatic antioxidants. The enzymatic antioxidants mainly consist of superoxide dismutases (SODs), catalase (CAT), heme oxidase (HO-1), glutathione S-transferases pi (GST-pi), NAD(P)H quinoneoxidoreductase (NQO1), glutamate-cysteine ligase modifier subunit (GCLM), glutathione peroxidases (GPXs), and reductase (GR) [7–9]; the endogenous nonenzymatic antioxidants include ascorbic acid (vitamin C), glutathione (GSH), reduced nicotinamide-adenine dinucleotide phosphate (NADPH), and *α*-tocopherol (the most active form of vitamin E) [10].

A growing number of studies have fully illustrated that the nuclear factor erythroid 2-related factor 2 (Nrf2) functions as the master regulator in the antioxidant stress pathway in mammal cells. Under basal conditions, NRF2 physically binds to the negative regulator Kelch-like ECH2-associated protein 1 (Keap1) for ubiquitination and proteasomal degradation within the cytoplasm, thus limiting Nrf2 activation (translocation to the nuclear) [11, 12]. However, under oxidative circumstances, Nrf2 is released from Keap1 and translocate into the nucleus, where it binds to antioxidant response elements (AREs), thus activating transcription of its target genes encoding phase II metabolizing enzymes and antioxidases, as well as molecular chaperones and anti-inflammatory factors [13–15]. Therefore, the Nrf2-ARE pathway has been one of the most important endogenous antioxidant stress pathway discovered to date. Furthermore, there has been evidence that Nrf2-deficient mice developed ocular pathology similar to cardinal features of human AMD and deregulated autophagy; there is likely a mechanistic link between oxidative injury and inflammation [16–20].

Tribulus terrestris (TT) is a plant that belongs to the family of Zygophyllaceae, and it is used individually as a single therapeutic agent or as a prime or subordinate component of many compound formulations and food supplements (health food) in China. The dry fruits of TT, as a traditional herb medicine (commonly named “Ji Li”), have been used for more than thousands of years in China within the context of protecting the liver, activating blood circulation, improving eyesight, and relieving itching (Chinese Pharmacopoeia, 2015 version). Modern studies have shown that TT has various pharmacological effects including anti-inflammatory, antioxidant, antibacterial, antiaging, and antitumor activities [21, 22]. TT has been used in clinical therapy, especially for the prevention and treatment of cardiovascular diseases, eye diseases, improving male sexual function, and diabetes [23–25]. The chemical constituents of the fruit of TT mainly include saponins, flavonoids, and alkaloids (polysaccharides, amino acids, and vitamins); the studies have shown that saponins and flavonoids in TT mainly contribute to its pharmacological activities.

For the first time, our present study investigates the underlying mechanisms of Tribulus terrestris (TT), which enables it to protect and restore the human retinal pigment epithelial cells (ARPE-19) against oxidative stress. Our results revealed that Tribulus terrestris (TT) has a significant antioxidant and cytoprotective effects on H_2_O_2_-treated ARPE-19 cells functioning through the PI3K/Akt-Nrf2 signaling pathway.

## 2. Materials and Methods

### 2.1. Cells and Cell Culture

Human RPE cell line (ARPE-19) was obtained from ATCC (American Type Culture Collection). Cells were cultured in GIBCO Dulbecco's Modified Eagle Medium. The medium was supplemented with 10% fetal bovine serum, 100 *μ*g/mL of streptomycin, and 100 U/mL of penicillin at 37°C with 5% CO_2_ in a humidified atmosphere; and the medium was changed every other day.

### 2.2. Preparation of the Ethanol Extracts of Tribulus terrestris (EE-TT)

First, Chinese herbal medicine of Tribulus terrestris (TT, dry fruits from the plant of *Tribulus terrestris* L. in China) 20 g was extracted by refluxing at 80°C in 200 mL of 70% ethanol and then the filtrate was collected twice for 1.5 hours each time. Then, the combined filtrate was adsorbed by the macroporous adsorbed resin column and then eluted with water and 95% ethanol in turn. Finally, 1.36 g of ethanol extract from Tribulus terrestris (EE-TT) was obtained by collecting of 95% ethanol eluted solution and recovering ethanol under the reduced pressure.

### 2.3. Cell Viability Assay and Oxidative Injury Model in ARPE-19 Cells

ARPE-19 cells viabilities were evaluated using 3-(4,5-dimethylthiazol-2-yl)-5-(3-carboxymethoxyphenyl)-2-(4-sulfophenyl) -2H-tetrazolium (MTS) reagent according to the manufacturer's instruction (Promega, USA). First, cells were plated in 96-well microplate with 2 × 10^4^ cells/well. Then, the cells were treated with individual concentration of H_2_O_2_ or ethanol extracts of Tribulus terrestris (EE-TT) for 24 h; or the cells were treated with 1 mM H_2_O_2_ for 24 h then followed by another 24 h exposure to the individual concentration of EE-TT; or the cells were treated with individual concentration of EE-TT for 4 h then followed by another 24 h exposure to 1 mM H_2_O_2,_ respectively. 5 mg/mL MTS solution was added (20 *μ*L/well), and the cells were incubated for 4 h at 37°C. Last, the absorbance was measured at 492 nm by a microplate reader (MD Emax Plus, USA). All experiments were performed in triplicate, and in each experiment, a minimum of three wells per treatment were used. The viability of ARPE-19 cells in each well was presented as the percentage of control cells (nontreated normal cells).

### 2.4. Apoptosis Assay of ARPE-19 Cells

The Annexin V-FITC/Propidium Iodide (PI) double-staining assay was used to detect cell apoptosis in this study. ARPE-19 cells were grown on a six-well plate at 1 × 10^5^ cells/well, and cells were either treated with or without different concentrations of TT for 24 h at 37°C after treatment with 1 mM H_2_O_2_ for 24 h at 37°C with 5% CO2. The cells were washed twice and collected with PBS (137 mM NaCl, 2.7 mM KCl, 4.3 mM Na_2_HPO_4_, and 1.4 mM KH_2_PO_4_). The cells were trypsinized and stained with annexin V-FITC and PI (from annexin V-FITC apoptosis detection kit, Nanjing Jiancheng Biology Engineering Research Institute, Nanjing, China) according to the manufacturer's protocol. The fluorescence intensity was measured by flow cytometry (FACSCalibur, BD Biosciences, San Diego, CA, USA). The cells were divided into viable cells (annexin V−/PI−), early apoptotic cells (annexin V+/PI−), late apoptotic cells (annexin V+/PI+), and necrotic cells (annexin V−/PI+). The stained cells were then analyzed with Cell-Quest software.

### 2.5. Measurement of Intracellular Reactive Oxygen Species (ROS)

The activities of ROS in ARPE-19 cells were detected by reactive oxygen species assay kit (Nanjing Jiancheng Biological Research Institute, China) following the manufacturer's directions. The cells were incubated with 10 *μ*M DCFH-DA reagent from the kit for 30 min at 37°C before being washed and suspended in PBS at 1 × 10^6^ cells/mL. Then, the cells were analyzed using flow cytometry at excitation and emission wavelengths of 488 and 525 nm, respectively. The untreated cells served as the control sample. The results were expressed as the mean of fluorescence intensity (MFI) of dichlorofluorescein (DCF) [26].

### 2.6. Measurement of Intracellular Superoxide Dismutase (SOD) Activity

The activities of SOD in ARPE-19 cells were detected using a superoxide dismutase assay kit (Nanjing Jiancheng Biological Research Institute, China). First, ARPE-19 cells were pretreated with 1 mM H_2_O_2_ for 24 h; or followed by a 24 h exposure to ethanol extracts of Tribulus terrestris (EE-TT). Then, the cells were repeatedly frozen and thawed several times in order to make sure the cells were thoroughly broken. Lastly, activities of SOD in ARPE-19 cells were detected to assess the antioxidative capacities (unit/per mg protein) using the superoxide dismutase assay kit following the manufacturer's directions [27].

### 2.7. Total Antioxidant Capacity Assay

Ferric reducing antioxidant power (FRAP) assay is a reliable and simple method for evaluating the inhibiting ability of antioxidants in the Fenton reaction [27]. The experiment was performed according to the protocol of the FRAP assay kit (Beyotime, Shanghai, China). The values of absorbance-concentrations of FeSO_4_ were used to make a standard curve, and the total antioxidant capacity was calculated as the concentration of FeSO_4_. The certain concentrations of positive antioxidant control Trolox (an analogue of vitamin E) or the ethanol extracts of Tribulus terrestris (EE-TT) were added in the reaction system, respectively. The reaction solution was incubated at 37°C for 3–5 minutes, and the value of absorbance was detected at 595 nm. The total antioxidant capacity of EE-TT or Trolox was calculated according to the standard curve of FeSO_4_.

### 2.8. Real-Time Quantitative PCR

Total RNA was extracted from ARPE-19 cells using TRIzol Reagent (Invitrogen, USA), and the cDNA was synthesized using GoScript™ Reverse Transcription System (Promega, USA) from 2 *μ*g RNA for each sample. Real-time PCR was performed using a GoTaq® qPCR Master Mix (Promega, USA) as follows: 20 *μ*L reaction solution contained 10 *μ*L SYBR Mix, 0.4 *μ*L sense and 0.4 *μ*L antisense primers solution (from 10 *μ*M), 1 *μ*L diluted cDNA, and 8.2 *μ*L nuclease-free water. The primer sequences used for real-time quantitative PCR are shown in Table 1 below. The subsequent data analysis was performed using MxPro™ QPCR Software followed by comparative quantification real-time PCR. The relative change in gene expression was determined according to the 2^−*ΔΔ*CT^ formula in which gene expression was normalized to the expression of *β*-actin (*ACTB*) in treated and untreated control samples.

### 2.9. Western Blot Analysis

The total proteins of ARPE-19 cells with or without treatments were collected and lysed in a buffer containing 50 mM Tris-HCl (pH 7.5), 150 mM NaCl, 2 mM EDTA, 1% Triton, 1 mM PMSF, and protease inhibitor cocktail on ice with rotation of 30 min. In order to examine Nrf2 expression, nuclear proteins or cytoplasmic proteins were isolated using a Bioepitope Nuclear Kit or Cytoplasmic Extraction Kit (Wanleibio, China) according to the protocol. The protein lysates (40 *μ*g of each sample) were separated by 10% or 12% sodium dodecyl sulfate- (SDS-) polyacrylamide gel electrophoresis and transferred to polyvinylidene difluoride (PVDF) membranes, which were blocked with primary and secondary antibodies according to the method we published previously [27]. The primary antibodies used in this study are as follows: Bcl2, Bax, cleaved caspase-3, cleaved caspase-9, NRF2, histone H3, and *β*-actin (1 : 500 or 1 : 1000, from Wanleibio, China); HRP-conjugated anti-mouse secondary antibodies (1 : 5000, from Wanleibio, China) were also used. The bands were visualized using an ECL western blotting detection kit (Beyotime, China) following the recommended procedures and the density of each band was measured using ImageJ software. The protein expression level of each molecular was normalized to *β*-actin protein (for total or cytoplasmic protein) or to histone H3 (for nuclear protein) expression level and compared with the untreated control sample, which was assigned to the value of 1 [28].

### 2.10. Statistical Analysis

All experiments were performed at least three times. Statistical analysis was carried out by the Statistical Product and Service Solutions (SPSS) software. Data are presented as mean ± SD (standard deviation). Student's *t*-test was used to calculate statistical significance between 2 groups. Statistical significance is denoted by the following: ^∗^*p* < 0.05, ^∗∗^*p* < 0.01, and ^∗∗∗^*p* < 0.001; or ^#^*p* < 0.05, ^##^*p* < 0.01, and ^###^*p* < 0.001.

## 3. Results

### 3.1. Tribulus terrestris Increased the Cell Viabilities in H_2_O_2_-Treated ARPE-19 Cells

**Figure 1:**
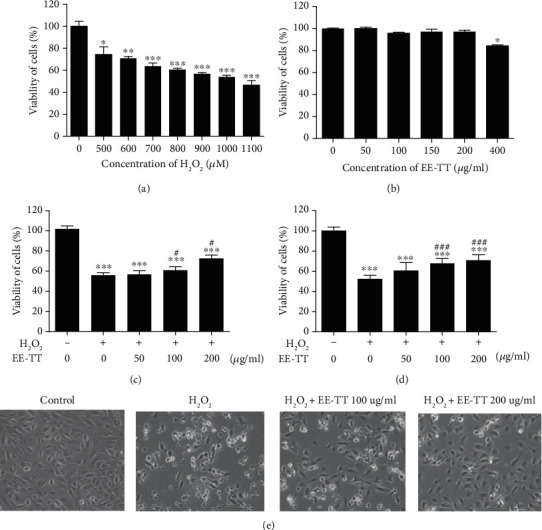
EE-TT increased the cell viabilities in H_2_O_2_-treated ARPE-19 cells. (a) ARPE-19 cells were treated with or without individual concentrations of H_2_O_2_, respectively, for 24 h. (b) ARPE-19 cells were treated with individual concentrations of EE-TT for 24 h. (c) ARPE-19 cells were pretreated with 1 mM H_2_O_2_ for 24 h, then cells were treated with individual concentrations of EE-TT for another 24 h. (d) ARPE-19 cells were pretreated with individual concentrations of EE-TT for 4 h, then cells were treated with 1 mM H_2_O_2_ for another 24 h. (a–d) The viability of cells was measured by MTS assay; the viability of cells was expressed as a percentage of the control group (nontreated cells). All data are shown as mean ± SD, ^∗^*p* < 0.05, ^∗∗^*p* < 0.01, and ^∗∗∗^*p* < 0.001 vs. the samples of control group (nontreated cells); ^#^*p* < 0.05 vs. the samples treated with H_2_O_2_ alone. (e) The cell morphology of ARPE-19 cells (c) was observed under an optic microscope (Nikon ECLIPSE TS100, Japan).

In this study, we used a H_2_O_2_-induced oxidative stress model in ARPE-19 cells. After 24 h treatment with the individual concentrations of H_2_O_2_, the cell viabilities were measured by MTS assay.  Figure 1(a) shows that H_2_O_2_ dose-dependently reduced the viability of ARPE-19 cells, and after treatment with 1000 *μ*M (1 mM) H_2_O_2_ for 24 h, the cell viability was about 54% compared with the untreated control sample. Accordingly, 1 mM H_2_O_2_ was used to induce oxidative injury in these cells for subsequent experiments. We also examined the potential toxicity of ethanol extracts of Tribulus terrestris (EE-TT) to ARPE-19 cells and found that there was no significant change in cell viability after 24 h incubation with EE-TT at 50, 100, 150, and 200 *μ*g/mL (Figure 1(b)). However, EE-TT at higher concentration (400 *μ*g/mL) had a toxic effect on ARPE-19 cells with a significant decrease in cell viability (Figure 1(b)). Consequently, EE-TT at a concentration of 100 or 200 *μ*g/mL was applied in subsequent experiments. Next, we observed that EE-TT concentration dependently increased cell survival rate in ARPE-19 cells after cells were exposed to 1 mM H_2_O_2_ for 24 h (Figure 1(c)), and treatment with 200 *μ*g/mL EE-TT for 24 h significantly increased cell viability from 55.8% to 72.6% compared with H_2_O_2_ only treated control sample (Figure 1(c)). In addition, we also observed that EE-TT concentration dependently increased cell survival rate in ARPE-19 cells when pretreatment with EE-TT for 4 h before cells were exposed to 1 mM H_2_O_2_ for 24 h (Figure 1(d)), pretreatment with 200 *μ*g/mL EE-TT for 4 h significantly increased cell viability from 52.1% to 70.5% compared with H_2_O_2_ only treated control sample (Figure 1(d)). The cell morphology of ARPE-19 cells (Figure 1(c)) was also observed under an optic microscope (Figure 1(e)). After H_2_O_2_ treatment, ARPE-19 cells became round, smaller, and the brightness of the cells darkened; some cell fragments can be observed under the microscope. While after exposure to EE-TT for 24 h, the number of damaged cells decreased significantly. Taken together, these data collectively indicated that EE-TT could protect and rescue ARPE-19 cells against H_2_O_2_-induced oxidative injury.

### 3.2. The Antiapoptotic Effects of Tribulus terrestris on Oxidative Stressed ARPE-19 Cells

**Figure 2:**
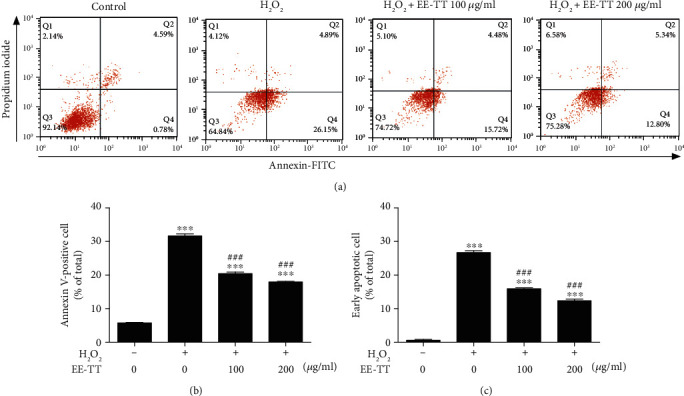
EE-TT inhibited H_2_O_2_-induced apoptosis in ARPE-19 cells. (a) ARPE-19 cells with or without H_2_O_2_ treatment for 24 h, then cells were treated with or without ethanol extracts of Tribulus terrestris (EE-TT) (100 or 200 *μ*g/mL) for another 24 h, the cells were stained with annexin V-FITC/PI double staining and analyzed by flow cytometry. The percentage of cells in each quadrant is presented. (b) The quantification of annexin V-FITC-positive cells (Q2 + Q4) was calculated for each group cells and is shown in the bar graph. (c) The quantification of early apoptotic cells (Q4) was calculated for each group cells and is shown in the bar graph. The data in (b) and (c) are shown as mean ± SD, ^∗^*p* < 0.05, ^∗∗^*p* < 0.01, ^∗∗∗^*p* < 0.001 vs. the samples of control group (nontreated cells); ^#^*p* < 0.05, ^##^*p* < 0.01, ^###^*p* < 0.001 vs. the samples treated with H_2_O_2_ alone.

To investigate whether Tribulus terrestris protects against H_2_O_2_-induced apoptosis, ARPE-19 cells were incubated with 1 mM H_2_O_2_ for 24 h and were then exposed to 100 or 200 *μ*g/mL EE-TT for 24 h. Cells were double stained with Annexin V-FITC/PI, and the percentage of apoptotic cells was determined with flow cytometry assay. The distribution of population of annexin V-FITC/PI staining cells is shown in Figure 2(a); the percentage of apoptotic cells (annexin V-FITC positive cells) or early apoptosis cells (annexin V-FITC positive/PI negative cells) was calculated and the results are presented in Figures 2(b) and 2(c), respectively. The results show that treatment with 200 *μ*g/mL EE-TT on ARPE-19 cells significantly reduced the percentage of apoptotic cells from 31.67% to 18.01% when compared with H_2_O_2_ alone treated sample, while it reduced the percentage of early apoptotic cells from 26.72% to 12.35% when compared with H_2_O_2_ alone treated sample. These results show that EE-TT could effectively protect and rescue ARPE-19 cells from H_2_O_2_-induced apoptotic cell damage.

**Figure 3:**
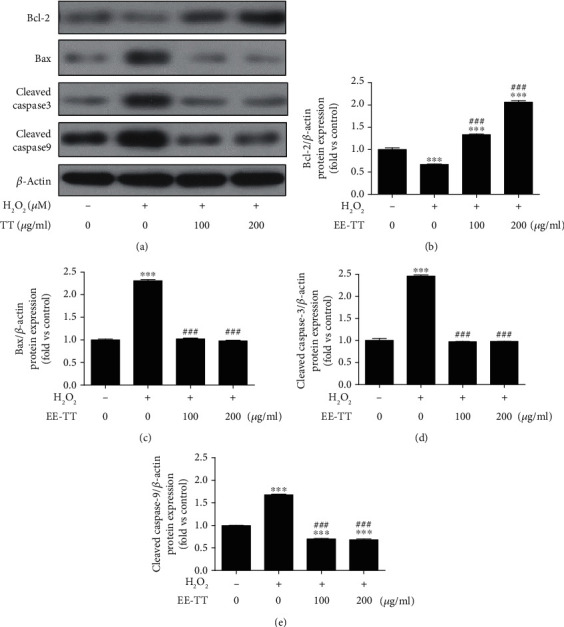
Effects of EE-TT on the protein expression levels of Bcl-2, Bax, caspase-3, and caspase-9 in H_2_O_2_-treated ARPE-19 cells. ARPE-19 cells were pretreated with or without 1 mM H_2_O_2_ for 24 h, followed by a 24 h exposure to ethanol extracts of Tribulus terrestris (EE-TT) (100 or 200 *μ*g/mL). (a) The cell lysates of control (nontreatment), H_2_O_2_-treated alone, or H_2_O_2_ plus EE-TT-treated samples were analyzed by Western blot assay with the antibodies of Bcl-2, Bax, cleaved caspase-3, cleaved caspase-9, and *β*-actin, respectively. Quantification data of these protein expression levels were measured and normalized to *β*-actin expression levels. The fold changes vs. control sample (nontreatment) were presented in the bar graph from (b–e), respectively. Data are shown as mean ± SD, ^∗^*p* < 0.05, ^∗∗^*p* < 0.01, ^∗∗∗^*p* < 0.001 vs. the nontreatment control sample; ^#^*p* < 0.05, ^##^*p* < 0.01, ^###^*p* < 0.001 vs. the sample with H_2_O_2_ treatment alone, *n* = 3.

Several studies have reported that H_2_O_2_-induced ARPE-19 cells apoptosis is related to the mitochondrial apoptotic signaling which involves the proapoptotic protein Bax, the antiapoptotic protein Bcl-2, and the downstream protein caspase families [29, 30]. We thus investigated and confirmed the possible mechanisms of the antiapoptotic effect of Tribulus terrestris on H_2_O_2_-treated ARPE-19 cells. The protein expression levels of Bcl2, Bax, caspase-3, and caspase-9 were measured by Western blot assay in H_2_O_2_-treated ARPE-19 cells followed by exposure to EE-TT for 24 h (Figure 3(a)). The fold changes of these protein expressions were calculated and presented in Figures 3(b)–3(e) bar graph. The results show that treatment with 200 *μ*g/mL EE-TT on ARPE-19 cells decreased the upregulated Bax, cleaved caspase-3, and cleaved caspase-9 protein expression levels induced by H_2_O_2_ around 60%, and meanwhile, treatment with 200 *μ*g/mL EE-TT on ARPE-19 cells increased the downregulated Bcl-2 protein expression levels induced by H_2_O_2_ about 207%. These results indicate that EE-TT protects and restores ARPE-19 cells from H_2_O_2_-induced apoptotic cell damage through Bcl2/Bax/caspases associated with signaling pathways.

### 3.3. Tribulus terrestris Affects H_2_O_2_-Induced Intracellular ROS and SOD Activities in ARPE-19 Cells

**Figure 4:**
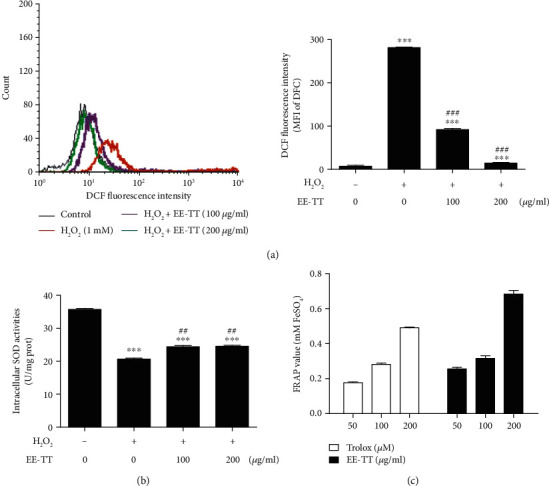
EE-TT significantly reduced the ROS activity while increasing SOD activity in H_2_O_2_-treated ARPE-19 cells. ARPE-19 cells were pretreated with or without 1 mM H_2_O_2_ for 24 h, followed by a 24 h exposure to ethanol extracts of Tribulus terrestris (EE-TT): (a) The intracellular ROS activities were measured and analyzed by a flow cytometer. ROS activity of each sample was presented by the mean of fluorescence intensity (MFI) of DCF; (b) The activities of intracellular SOD were measured by a SOD activity assay kit. The SOD levels were normalized against per milligram protein. (c) Comparison of total antioxidant capacity of EE-TT and Trolox (an analogue of vitamin E, a positive antioxidant control). All data are shown as mean ± SD (*n* = 3). ^∗^*p* < 0.05, ^∗∗^*p* < 0.01, ^∗∗∗^*p* < 0.001 vs. the samples of control group (nontreated cells); ^#^*p* < 0.05, ^##^*p* < 0.01, ^###^*p* < 0.001 vs. H_2_O_2_ only treated group.

Many studies have demonstrated that oxidative stress leads to reactive oxygen species (ROS) production beyond the limits of clearance in vivo and causes oxidation and antioxidant system imbalance, which results in functional and morphological impairments of retinal pigment epithelium (RPE), endothelial cells, and retinal ganglion cells [31]. In addition, superoxide dismutase (SOD) is one of the most important antioxidant enzymes of the intracellular antioxidant defense system. SOD can remove oxygen-free radicals and protect cells from oxidative injury and the level of SOD activity reflects the cellular antioxidant ability. Therefore, we were interested to investigate whether EE-TT could restore the oxidative injury of ARPE-19 cells induced by H_2_O_2_-treatment. In Figures 4(a) and 4(b), ROS and SOD activities were measured in ARPE-19 cells with EE-TT treatment after exposure to H_2_O_2_ for 24 h. In Figure 4(a), the data shows that H_2_O_2_ induced a clear increase of intracellular ROS activities compared with non-H_2_O_2_-treated sample (mean of fluorescence intensity, MFI, from 8.4 to 281); treatment with EE-TT remarkably decreased the upregulated ROS activities induced by H_2_O_2_ in a dose-dependent manner (mean of fluorescence intensity, MFI, from 281 to 93, 15, respectively). We also observed that H_2_O_2_ treatment significantly reduced 42% of the intracellular SOD activities compared with non-H_2_O_2_-treated sample; and treatment with 100 or 200 *μ*g/mL EE-TT noticeably enhanced the downregulated SOD activities induced by H_2_O_2_ (Figure 4(b)).

In addition, a comparison of antioxidant activity between EE-TT and vitamin E (a positive antioxidant control) was performed to determine the antioxidant capacity of EE-TT itself (Figure 4(c)); the results show that the antioxidant capacity of 200 *μ*g/mL EE-TT is higher than that of 200 *μ*M Trolox (an analogue of vitamin E, a positive antioxidant control), while both results showed the similar levels of cell viability after treating the cells for 24 h on oxidative stressed ARPE-19 cells (data not shown). In sum, the above results show that ethanol extracts of Tribulus terrestris (EE-TT) could effectively affect the oxidation and antioxidant imbalanced system induced by H_2_O_2_ through the regulations of both activities of ROS and SOD in ARPE-19 cells.

### 3.4. The Activation of Tribulus terrestris on the mRNA Expressions of Nrf2 and Its Target Genes in ARPE-19 Cells

**Figure 5:**
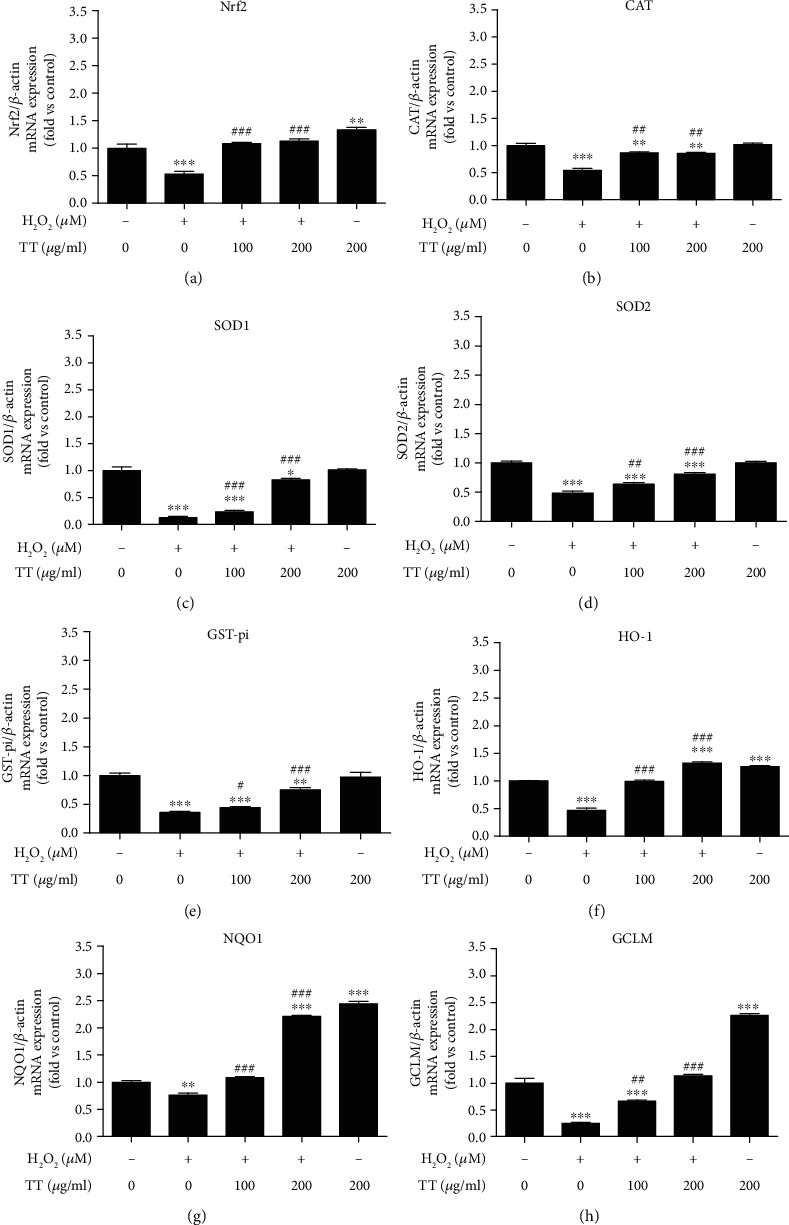
Effects of EE-TT on mRNA expression levels of rodex-related genes in H_2_O_2_-treated ARPE-19 cells. ARPE-19 cells were pretreated with or without 1 mM H_2_O_2_ for 24 h, followed by a 24 h exposure to ethanol extracts of Tribulus terrestris (EE-TT). The mRNA expression levels of Nrf2 (a), CAT (b), SOD1 (c), SOD2 (d), GST-pi (e), HO-1 (f), NQO1 (g), and GCLM (h) for each sample were measured by real-time PCR; the fold changes of each gene are shown in the bar graph compared with the control sample (nontreated cells, value of 1). The data are shown as mean ± SD, ^∗^*p* < 0.05, ^∗∗^*p* < 0.01, ^∗∗∗^*p* < 0.001 vs. the control sample (non-treated cells); ^#^*p* < 0.05, ^##^*p* < 0.01, ^###^*p* < 0.001 vs. the sample with H_2_O_2_ only treated sample.

As demonstrated above, Tribulus terrestris protected ARPE-19 cells against oxidative stress by inhibiting H_2_O_2_-induced ROS production and apoptosis, thereby noticeably improved the cell viability. An increasing number of studies have reported that the activation of the transcription factor NRF2 (nuclear factor erythroid 2-related factor 2) signaling pathway may be involved in the antioxidant responses against the oxidative stress in ARPE-19 cells, especially through regulation of its target genes which encoding phase II enzymes including HO-1, SOD, NQO1, and GCLM [32–34]. We then investigated whether ethanol extracts of Tribulus terrestris (EE-TT) could regulate the mRNA expression level of Nrf2 and its target genes under both conditions with or without H_2_O_2_-treatment. In Figure 5, the ARPE-19 cells were pretreated with or without 1 mM H_2_O_2_, then cells were treated with or without EE-TT for 24 h; the mRNA levels of Nrf2, CAT, SOD1, SOD2, GST-pi, HO-1, NQO1, and GCLM were measured by real-time PCR. As shown in Figure 5, treatment with 1 mM H_2_O_2_ alone for 24 h significantly decreased the Nrf2, CAT, SOD1, SOD2, GST-pi, HO-1, NQO1, and GCLM mRNA expression levels compared with the untreated control sample. Also, EE-TT treatment noticeably restored all of these enzymes or proteins mRNA expression levels in H_2_O_2_-treated ARPE-19 cells. Furthermore, treatment with 200 *μ*g/mL EE-TT alone on ARPE-19 cells for 24 h remarkably upregulated Nrf2, HO-1, NQO1, and GCLM mRNA expression levels about 1.33, 1.26, 2.44, and 2.25 folds, respectively. These results suggest that EE-TT may activate Nrf2 signaling through the upregulation of its mRNA expression levels.

### 3.5. Tribulus terrestris Upregulated the mRNA Expression Levels of Nrf2, HO-1, NQO1, and GCLM through PI3K/Akt Signaling Pathway in ARPE-19 Cells

**Figure 6:**
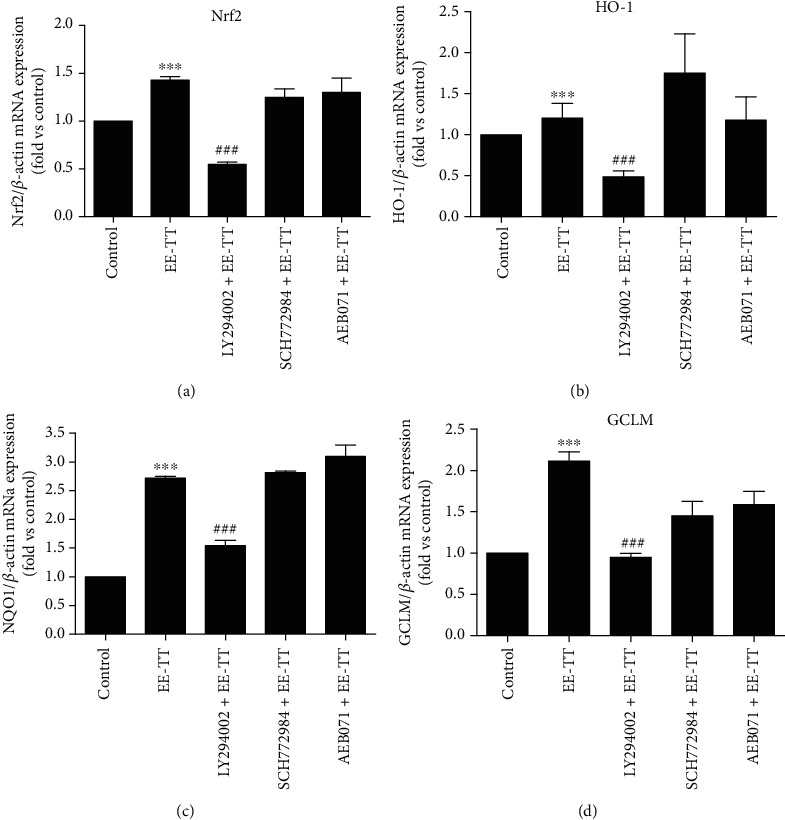
Inhibitor of PI3K/Akt signaling pathway blocked the upregulation of mRNA expression levels of Nrf2, HO-1, NQO1, and GCLM by EE-TT treatment in ARPE-19 cells. ARPE-19 cells were pretreated with or without the inhibitors of 10 *μ*M LY294002, 0.25 *μ*M SCH772984, and 0.5 *μ*M of AEB071 for 1 h, followed by a 24 h exposure to 200 *μ*g/mL EE-TT. The mRNA expression levels of Nrf2 (a), HO-1(b), NQO1 (c), and GCLM (d) for each sample were measured by real-time PCR, and the fold changes of each gene are shown in the bar graph compared with the control sample (nontreated cells, value of 1). All data are shown as mean ± SD, ^∗^*p* < 0.05, ^∗∗^*p* < 0.01, ^∗∗∗^*p* < 0.001 vs. the control sample (nontreated cells); ^#^*p* < 0.05, ^##^*p* < 0.01, ^###^*p* < 0.001 vs. the sample with EE-TT treated only cells.

The previous studies have reported that the regulation or activation of Nrf2-ARE signaling may be through PI3K/Akt (phosphoinositide 3-kinase and AKT serine/threonine kinase 1), ERK/MAPK, or PKC signaling pathways [35–37]; we thus detected which signaling pathways are involved in the regulation of EE-TT on NRF2 and its associated antioxidant genes expressions. In Figure 6, ARPE-19 cells were treated with or without the inhibitors of PI3K/Akt (LY 294002), ERK1/2 (SCH772984), or PKC (AEB071) for 2 h before being exposed to 200 *μ*g/mL EE-TT for 24 h; the mRNA expression levels were measured by real-time PCR. The results demonstrate that pretreatment with the inhibitor of PI3K/Akt (LY294002) clearly eliminated the upregulation of mRNA expressions of Nrf2, HO-1, NQO1, and GCLM genes in ARPE-19 cells.

### 3.6. Tribulus terrestris Enhanced the Cell Viability of Oxidative Stressed ARPE-19 Cells through PI3K/Akt Signaling Pathway

**Figure 7:**
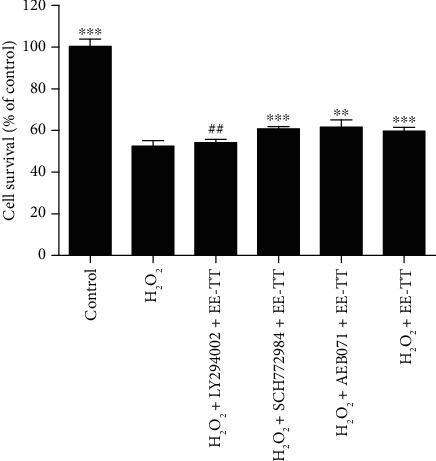
Tribulus terrestris increased the cell viability of oxidative stressed ARPE-19 cells through PI3K/Akt signaling pathway. ARPE-19 cells were treated with or without 1 mM H_2_O_2_ for 24 h, then cells were exposed to 200 *μ*g/mL EE-TT after 1 h treatment with the inhibitors of 10 *μ*M LY294002, or 0.25 *μ*M SCH772984, or 0.5 *μ*M of AEB071, respectively. The viability of cells was measured by the MTS assay and was expressed as a percentage of the control group (nontreated cells). All data are shown as mean ± SD, ^∗^*p* < 0.05, ^∗∗^*p* < 0.01, ^∗∗∗^*p* < 0.001*vs*. the sample with H_2_O_2_ treated only cells; ^#^*p* < 0.05, ^##^*p* < 0.01 vs. the sample with H_2_O_2_ and EE-TT treated cells.

In order to examine whether the inhibition of PI3K/Akt signaling pathway could also accordingly affect the cell survival of H_2_O_2_-treated ARPE-19 cells, in Figure 7, after ARPE-19 cells were treated with or without 1 mM H_2_O_2_ for 24 h, the cells were then pretreated with the inhibitors for 1 h, followed by treatment with 200 *μ*g/mL EE-TT for 24 h; the viability of cells was measured by MTS assay. As shown in Figure 7, the result indicates that pretreatment with the inhibitor of PI3K/Akt (LY 294002) signaling pathway significantly blocked the increased cell viability induced by EE-TT treatment.

### 3.7. Tribulus terrestris Activated the Nrf2 Protein Translocation to the Nuclear

**Figure 8:**
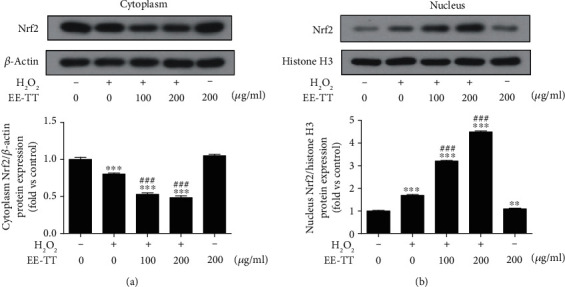
EE-TT promotes the nuclear translocation of Nrf2 in H_2_O_2_-treated ARPE-19 cells. ARPE-19 cells were pretreated with or without 1 mM H_2_O_2_ for 24 h, followed by a 24 h exposure to ethanol extracts of Tribulus terrestris (EE-TT). The results of Western blot analyses for protein abundance of Nrf2 in the cytoplasm (a) and nucleus (b) with quantification data are showed and the fold changes vs. control sample (nontreatment) are presented in the bar graph. Data are shown as mean ± SD, ^∗^*p* < 0.05, ^∗∗^*p* < 0.01, ^∗∗∗^*p* < 0.001 vs. the nontreatment control sample; ^#^*p* < 0.05, ^##^*p* < 0.01, ^###^*p* < 0.001 vs. the sample with H_2_O_2_ treatment alone.

To further investigate the antioxidant mechanisms of Tribulus terrestris on oxidative stressed ARPE-19 cells, we tested whether EE-TT could promote the Nrf2 translocation to the nucleus and activate the Nrf2 signaling. ARPE-19 cells were pretreated with or without 1 mM H_2_O_2_ for 24 h, followed by a 24 h exposure to EE-TT (100 or 200 *μ*g/mL), then the cytoplasm (a) or the nucleus protein (b) was analyzed using Western blot assay with the antibodies of Nrf 2, *β*-actin, or histone H3. First, as shown in Figure 8, treatment with 1 mM H_2_O_2_ alone for 24 h significantly decreased the cytoplasm Nrf2 protein level while increased the nuclear Nrf2 protein level. Furthermore, EE-TT remarkably promoted the Nrf2 protein translocation from cytoplasm to the nuclear in a dose-dependent manner. In addition, ARPE-19 cells treatment with 200 *μ*g/mL EE-TT alone for 24 h had no significant effect on the Nrf2 protein expressions neither in the cytoplasm nor in the nucleus.

## 4. Discussion

In recent years, there have been more and more reports about the relations between oxidative stress and ophthalmic diseases. Oxidative stress has been found to induce or accelerate pathological progression in some ophthalmic diseases (including dry eye, AMD, and DR) [6, 38, 39]. In fact, there are many studies that focus on examining the effects of oxidative stress on the development of AMD diseases using in vitro and in vivo experiments [34, 40, 41]. In addition, it has been reported in one clinical research that the serum level of total oxidant status (TOS, an oxidative stress parameter) was significantly increased by about 167% in 22 AMD patients when compared with that of a group of 25 healthy people, while the levels of two important antioxidant molecules (total thiol status (TTS) and the activity of paraoxonase 1(PON1)) were significantly decreased. Based on these results, it can be stated that increased oxidative stress and decreased antioxidant levels may have a synergistic role in AMD development [42]. Another clinical trial has evaluated the effects of several antioxidant agents on the progression of AMD and visual acuity. This trial was carried out using pharmacological doses, including *β*-carotene, vitamin C, vitamin E, zinc, and copper. Their data demonstrated that the administration of antioxidants could result in a 25% risk reduction in advanced AMD progression and a 19% risk reduction in moderate vision loss within 5 years [43]. In light of these results, it has been demonstrated that the use of antioxidants for the treatment or adjuvant treatment of AMD disease is a prospective therapeutic treatment.

The main active ingredients of Tribulus terrestris (TT) are saponins, flavonoids, alkaloids, amino acids, and the others (such as glycosides, fatty acids, and vitamins) [44]. Many studies have shown that saponins and flavonoids in TT are the main contributors of its pharmacological benefits including having aphrodisiac, anti-inflammatory, antimicrobial, and antioxidant abilities [45]. Although natural saponins and flavonoids usually have low oral bioavailability (the blood concentration is from nanomolar to micromolar), there are studies that have shown that oral administration of saponins or flavonoids could significantly improve ocular symptoms of certain retinal diseases (such as diabetic retinopathy and retinal vein occlusion), which were confirmed by the in vivo animal experiments or the clinical trials [46, 47]. These results suggest that some natural products or their metabolites may function by circulating blood into the retina. Saponins are one of the primary components identified in TT and, so far, about 108 kinds of steroidal saponins have been isolated from TT. Among these, protodioscin and protogracillin are thought to confer TT's unique biological activities [46]. The main pharmacological activities of saponins in TT include its effects on lowering blood pressure, lowering blood lipid, and protecting the cardiovascular system against myocardial ischemia [47–49]; improving the apoptosis rate of ischemic brain nerve cells, protecting brain nerve, enhancing memory and antidepressant effect, reducing blood glucose and diabetes-induced apoptosis of optic nerve cells in diabetic mice [50–53]. Some studies have reported that the saponins from TT could improve primary mice retinal ganglion cells (RGCs) survival when cultured in vitro [38, 54]. Today, saponins from TT are used for sequelae of cerebrovascular disease and coronary heart disease treatments [55–57]. As the main active ingredient of TT, the main flavonoids extracted from Tribulus terrestris include quercetin, kaempferol, isorhamnetin, and their derivatives according to their skeleton structures [36]. In recent years, the natural flavonoids from many edible plants (such as vegetables, fruits, and beans) or from traditional herb medicines have become a subject of interest due to their potent antioxidant capacity and low toxicity, which in return, makes them highly effective as potential therapeutic agents. In natural flavonoids, quercetin appears to be the subject of the most studies in different tissues or cell types, and the protective effects of quercetin against oxidative-induced damages in ARPE-19 cells have also been reported [34, 57] with the activation of Nrf2 pathway and its target genes implicated in antioxidant defense. Kaempferol is a member of the flavonols family, widely found in many edible plants and traditional herb medicines (such as chrysanthemum, astragalus mongholicus, ginkgo leaf, and dry raspberry). In 2018, our group reported that kaempferol has stronger antioxidant activity than that of lutein and resveratrol. Our data illustrated that the nanomole concentration of kaempferol has significant effects on improving the oxidative damage of ARPE-19 cells or rat RPE cells tested by in vitro or in vivo experiments. We also demonstrated that treatment with kaempferol significantly reduced the upregulated ROS activity while increasing the SOD activity, as well as inhibited the apoptosis by regulating the Bcl2, Bax, and caspase-3 proteins expressions in H_2_O_2_-treated ARPE-19 cells [27]. Isorhamnetin is also a common flavonoid found in nature. The protective effects of isorhamnetin on oxidative stressed ARPE-19 cells were also reported by Liao et al. [53] in 2009. They found that isorhamnetin pretreatment significantly increased the phosphorylation of phosphoinositide 3-kinase (PI3K) and AKT serine/threonine kinase 1 (Akt) in ARPE-19 cells exposed to H_2_O_2_ compared with cells treated with H_2_O_2_ alone, suggesting that the protective effects of isorhamnetin on oxidative stressed ARPE-19 cells might be through PI3k/Akt associated signaling pathway.

Numerous reports have proved that Nrf2 is an emerging regulator of cellular resistance to oxidative stress. Some natural products (such as apigenin, quercetin, gypenosides, genipin, rhizoma paridis, escin, and astaxanthin) have also been shown to have protective effects against the oxidative stressed ARPE-19 cells associated with Nrf2 signaling [28, 57–63]. In our study, we found that treatment with 1 mM H_2_O_2_ for 24 h significantly induced a serious oxidative cell damage and apoptosis in ARPE-19 cells (Figures 1 and 2). In the meantime, the mRNA expressions of Nrf2 and its target genes encoding phase II metabolizing enzymes and antioxidases (such as CAT, HO-1, SOD1, SOD2, GST-pi, NQO1, and GCLM) were also remarkably decreased by H_2_O_2_ when compared with control cells (Figure 5). These results are very similar to those of another group's work [59] in which they also used the high concentration (750 *μ*M) of H_2_O_2_ to induce oxidative stressed model in ARPE-19 cells. Although, in the studies of another two groups who investigated the protective effects of apigenin [28] or quercetin [57] on tBHP or H_2_O_2_-induced oxidative stressed model in ARPE-19 cells, treatment with 200 *μ*M H_2_O_2_ alone did not change the Nrf2 mRNA expression. Though, the nuclear Nrf2 protein expression was increased and the mRNA expression levels of phase II enzymes and antioxidases (such as HO-1, NQO1, and GCL) were upregulated as well. These results suggest that the Nrf2 signaling was activated [57]. Combined with all the above results, it is clear that the high concentration of H_2_O_2_ may induce serious oxidative stress and thereby inhibiting the Nrf2 mRNA expression and Nrf2 activation. However, treatment with a low concentration of H_2_O_2_ (200 *μ*M) may induce mild oxidative stress and could induce the activation of Nrf2, resulting in the increase of mRNA and protein expressions of phase II enzymes and antioxidases. In our study, treatment with EE-TT in the absence of H_2_O_2_ also increased the mRNA expressions of Nrf2 and the phase II enzymes and antioxidases (including NQO1, GCLM, and HO-1, but not for CAT, SODs, and GST-pi) (Figure 5). These results are similar to those of the other experiments, which treatment ARPE-19 cells with apigenin [28], genipin [60], escin [62], or astaxanthin [63], respectively. The underlying mechanisms involved in this regulation might be cell type or oxidant type dependent [7, 26, 28, 37, 49, 64]. Furthermore, treatment with EE-TT significantly increased the translocation of Nrf2 to the nucleus, as well as restored the downregulated mRNA expression levels of Nrf2 and phase II enzymes and antioxidases in H_2_O_2_-treated ARPE-19 cells (Figures 5 and 8). This suggests that the components of EE-TT could affect Nrf2-ABE signaling not only under normal condition but also under the oxidative stress condition.

In summary, our study shows for the first time that ethanol extracts of Tribulus terrestris (EE-TT) exhibit potent protective effects on ARPE-19 cells against H_2_O_2_-induced oxidative injury, which are associated with its antioxidant effects dependent on the activation of PI3K/Akt -Nrf2 signaling. Our current study strongly demonstrates that Tribulus terrestris could be a safe preventive and therapeutic option for patients with AMD or other retinal diseases patients.

## Figures and Tables

**Table 1:** The primer sequences for real-time quantitative PCR.

Gene name	Primer sequences	
	Forward (5′-3′)	Reverse (5′-3′)
*NRF2*	TTCTCCCAATTCAGCCAGCC	ACGTAGCCGAAGAAACCTCAT
*CAT*	ACTGTTGCTGGAGAATCGGG	AGGACGTAGGCTCCAGAAGT
*SOD1*	TGAAGGTGTGGGGAAGCATT	AGTCTCCAACATGCCTCTCTTC
*SOD2*	GCATCAGCGGTAGCACCA	TGGGCTGTAACATCTCCCTTG
*GST-pi*	AGACCAGATCTCCTTCGCTG	AGGTTCACGTACTCAGGGGA
*HO-1*	ACTGCGTTCCTGCTCAACAT	GGGCAGAATCTTGCACTTTGTT
*NQO1*	GGTTTGGAGTCCCTGCCATT	CCTTCTTACTCCGGAAGGGTC
*GCLM*	CAGCGAGGAGGAGTTTCCAG	GAACAGGCCATGTCAACTGC
*ACTB*	CATGTACGTTGCTATCCAGGC	CTCCTTAATGTCACGCACGAT

## Data Availability

Please contact Dr. Donglei Zhang if somebody needs the data or information about this manuscript. Please e-mail to: zhangdonglei@huh.edu.cn.
